# Trauma and parental separation experienced before age 18 as predictors of psychopathology in youngsters from the community assessed longitudinally for over 9 years

**DOI:** 10.1192/j.eurpsy.2026.10337

**Published:** 2026-07-31

**Authors:** C. L. Vandeleur

**Affiliations:** Psychiatry, Lausanne University Hospital, Prilly, Switzerland

## Abstract

**Aims:**

Although retrospective studies of adults found that mood and anxiety disorders may follow exposure to trauma, prospective longitudinal research on this topic in children and adolescents is still rare. The goal of this analysis was to assess the incidence of major depressive and anxiety disorders in offspring who experienced trauma or parental separation during childhood or adolescence, over almost a decade of follow-up.

**Methods:**

As part of a family study in the community of Lausanne, we directly interviewed 461 offspring before age 18, who subsequently undertook a second diagnostic interview. The mean age of the offspring at study intake was 12.8 (s.d. 3.4) years and the mean follow-up duration was 9.3 (s.d. 4.4) years. Traumatic events were defined as having been in an accident, being a victim of crime or physical/sexual abuse or having witnessed events experienced by others. The hazard risk of psychopathology in affected versus non affected offspring was determined using time-varying shared gamma-frailty models, adjusted for intra-familial correlation.

**Results:**

In total, 23 offspring reported premorbid trauma and 75 reported premorbid parental separation. Analyses revealed that offspring who had experienced early trauma reported an earlier onset of anxiety disorders than offspring who had not. In contrast, offspring who had experienced early parental separation did not report more anxiety or major depressive disorders over the follow-up than those who had not experienced parental separation.

**Conclusions:**

These findings underline the need for early intervention with offspring who experience early trauma to alleviate the development of future anxiety disorders.

**Disclosure of Interest:**

None Declared

